# Conventional type 1 dendritic cells (cDC1) in cancer immunity

**DOI:** 10.1186/s13062-023-00430-5

**Published:** 2023-11-01

**Authors:** Peng Liu, Liwei Zhao, Guido Kroemer, Oliver Kepp

**Affiliations:** 1 Centre de Recherche des Cordeliers, Equipe Labellisée par la Ligue Contre le Cancer, Université de Paris Cité, Inserm U1138, Institut Universitaire de France, Sorbonne Université, 75006 Paris, France; 2 Metabolomics and Cell Biology Platforms, Gustave Roussy Cancer Center, 94800 Villejuif, France; 3 Department of Biology, Institut du Cancer Paris CARPEM, Hôpital Européen Georges Pompidou, AP-HP, 75015 Paris, France

## Abstract

Cancer immunotherapy, alone or in combination with conventional therapies, has revolutionized the landscape of antineoplastic treatments, with dendritic cells (DC) emerging as key orchestrators of anti-tumor immune responses. Among the distinct DC subsets, conventional type 1 dendritic cells (cDC1) have gained prominence due to their unique ability to cross-present antigens and activate cytotoxic T lymphocytes. This review summarizes the distinctive characteristics of cDC1, their pivotal role in anticancer immunity, and the potential applications of cDC1-based strategies in immunotherapy.

## Introduction

Cancer has long been considered a cell-autonomous genetic disease, which occurs as a consequence of accumulating genomic mutations facilitating unrestricted growth and malignant dissemination. More recently it became clear that the evasion of malignant cells from immune destruction constitutes yet another important hallmark of cancer that can be targeted by clinical immuno-oncology. At this moment most immunotherapeutic approaches for the routine management of cancer are based on the (re)activation of cytotoxic T lymphocytes (CTLs) by means of monoclonal-antibodies that target immune checkpoints such as CTL associated protein 4 (CTLA-4) or programmed cell death protein 1 (PDCD1, best known as PD-1) and its ligand cluster of differentiation 274 (CD274, best known as PD-L1). The use of immune checkpoint inhibitors (ICI) has significant effects on overall survival in the adjuvant and neoadjuvant regimen of distinct malignant indications [1–3]. Nevertheless, the success of ICI monotherapy is limited to only a fraction of patients and depends on the expression of immune checkpoint molecules, the tumor mutational burden of the malignancy, as well as on the general immune tonus of the patient.

Additional therapeutic strategies that aim at reestablishing cancer immunosurveillance in combination with immune checkpoint blockade involve chemotherapy (chemoimmunotherapy), radiotherapy (radioimmunotherapy) and chemoradiotherapy (chemoradioimmunotherapy). Such approaches have shown success when the cytotoxic treatment induced immunogenic cell death (ICD) in cancer cells, which then act as an in situ vaccine that triggers adaptive anticancer immunity, hence sensitizing tumors for subsequent immunotherapy [4]. In an ideal scenario, such combination treatments elicit resilient immunological memory, which confers durable disease control [5–8]. ICD-associated cellular stress responses induce epigenetic shifts, alternative splicing event, the expression of conventionally silent coding sequences as well as specific post-translational modifications leading to alterations in the tumor proteome and facilitating the generation of non-mutational neoantigens [9]. Moreover, in the course of ICD, cancer cells emit a characteristic array of damage-associated molecular patterns (DAMPs), that act as adjuvants on innate pattern recognition receptors (PRRs) expressed by antigen-presenting cells (APCs) of the conventional dendritic cell (DC) type 1 (cDC1) [10–12]. The recruitment of such antigen presenting cells into the tumor bed is orchestrated by the specific temporal and spatial appearance of ICD-associated DAMPs, including the early release of adenosine triphosphate (ATP) and annexin A1 (ANXA1). ATP and ANXA1 ligate purinergic receptors of the purinergic receptor P2X 7 (P2RX7) type and formyl peptide receptor 1 (FPR1), respectively, thus facilitating the chemoattraction and homing of migratory cDC1s into the tumor bed, into the proximity of stressed and dying cancer cells [13–15]. Furthermore, surface-exposed calreticulin (CALR), which interacts with LDL receptor-related protein 1 (LRP1), serves as a de novo uptake signal and facilitates DC-mediated phagocytosis of tumor cells, hence resulting into the transfer of tumor-associated antigens into antigen-presenting cells [16–19]. The exodus of high mobility group box 1 (HMGB1) late in the course of ICD triggers Toll-like receptor 4 (TLR4)-mediated tumor antigen processing and ultimately drives DC maturation [20, 21].

Additional ICD-related immunostimulatory signaling comprises the release of tumor cell-derived genomic and mitochondrial DNA into the cytosol of cancer cells (or their uptake by antigen presenting cells present in the tumor microenvironment) that then induce the cyclic GMP-AMP synthase (CGAS)/stimulator of interferon response CGAMP interactor 1 (STING1) pathway, as well as the liberation of transcription factor A, mitochondrial (TFAM), which serves as a ligand for advanced glycosylation end-product specific receptor (AGER), thus further stimulating DC maturation [22, 23]. Robust type-1 interferon (IFN) responses in DC ultimately result in the C-X-C motif chemokine ligand 10 (CXCL10)-dependent recruitment of T lymphocytes and the onset of adaptive immune responses [24–27].

**Fig. 1:**
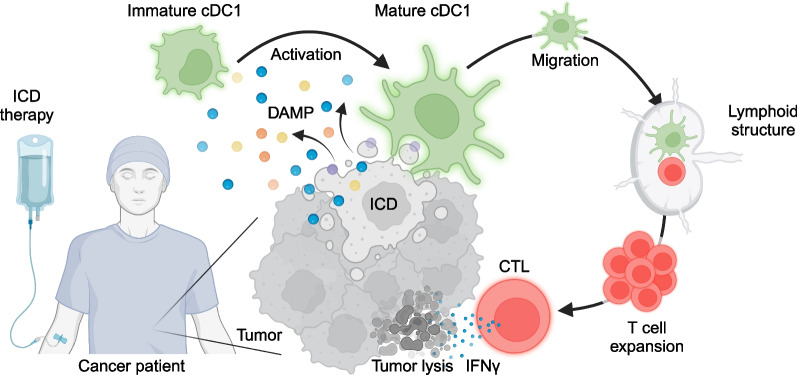
Immunogenic cell death-activated and cDC1-mediated anticancer immunity. ICD-inducing therapies have the ability to stimulate the antigenicity and adjuvanticity of malignant cells, via a viral mimicry that facilitates the emission of danger associated molecular patterns (DAMP) by the cancer cells which in turn lead to the recruitment and activation of professional antigen-presenting cDC1 dendritic cells into the tumor bed. Activated mature cDC1s can migrate to tertiary lymphoid structures or to draining lymph nodes for the education of cytotoxic T lymphocytes (CTL) that then engage in the destruction of residual or distant cancer cells (Created with BioRender.com)

Altogether, ICD stimulates the antigenicity and adjuvanticity of the tumor, thus inducing a sort of viral mimicry that facilitates the recruitment and activation of professional antigen-presenting cDC1 in the tumor bed. Activated cDC1s in turn can migrate to tertiary lymphoid structures within the tumor bed or to draining lymph nodes for the education of effector T cells that engage in the destruction of residual or distant cancer cells (Fig. 1).

## Definition of the cDC1 subset compared to other DC populations

ICD-relevant cDC1 belong to the group of conventional DC (cDC) which can be further subdivided into cDC1s and cDC2s that both express CD11c and MHC class II, knowing that additional DC subsets have been described in both mice and humans [28, 29].

In humans, cDC1 and cDC2 develop from myeloid progenitor pre-DC via precursor cells dubbed pre-cDC1 and pre-cDC2, respectively, whereas plasmacytoid DC (pDC) arise from the lymphoid lineage [28, 30–33]. The cDC2 population is heterogenous and can be further subdivided into DC2 and DC3 based on single-cell transcriptional profiles [30]. The development of the cDC1 subset depends on the activity of the transcription factors basic leucine zipper ATF-Like transcription factor 3 (BATF3), interferon regulatory factor 8 (IRF8) and inhibitor of DNA binding 2 (ID2) [34]. Moreover, the cDC1 subset can be formally distinguished from other DC subsets by virtue of specific surface markers, such as X-C motif chemokine receptor 1 (XCR1) and the C-type lectin domain containing 9A (CLEC9A) [35, 36]. Integrin alpha E, epithelial-associated (Itgae; best known as CD103) is commonly considered as an additional marker of mouse cDC1s, while thrombomodulin (THBD, also known as BDCA3 or CD141) is expressed on human cDC1s [37].

At the functional level, DC subsets are specialized in the response to different pathogens. cDC1s play a major role in mounting adaptive immune responses against intracellular pathogens such as viruses due to their ability to cross-present cellular antigens to CD8^+^ T cells. Thus, cDC1s play also a major role in antitumor immunity. cDC2 orchestrate immune responses to extracellular pathogens via the activation of CD4^+^ T helper cells. pDC produce type I IFNs in response to viral infection, although IFN-α/β production in cancer is often impaired [38].

Altogether, cDC1 can be distinguished from other DC subsets on several levels, namely their origin from the myeloid lineage, as well as the distinctive expression of surface markers. In addition, the migratory phenotype of cDC1 and their unique ability to induce CD8^+^ CTL responses make them indispensable for the onset of adaptive anticancer immunity in clinical settings.

## Essential impact of cDC1 in cancer immunotherapy

Despite the general scarcity of cDC1s, their overall abundance in the tumor is associated with increased objective response and overall survival in multiple human cancers [39, 40]. Moreover, cDC1s are crucial for antitumor immunity and the success of anticancer immunotherapy [41, 42] (recently reviewed in detail by Kvedaraite and Ginhoux) [33].

The impact of cDC1s on anti-tumor immunity has been demonstrated in studies employing cDC1-deficient Batf3^−/−^ mice and other models of cDC1 depletion. These models consistently showed that the lack of cDC1 was associated with the absence of CD8^+^ effector T cell recruitment, hence resulting in the failure of T cell-based immunotherapies including adoptive T cell transfer and immune checkpoint blockade. Reconstitution with activated DC from Batf3^+/+^ mice restored CD8^+^ effector T cell migration into the tumor bed. In sharp contrast, the lack of CD103^+^ cDC1s could not be compensated by other dendritic cell subsets or through BATF3-independent cytokine-induced cDC1 development [43–45]. Consistently, in a mouse melanoma model, the systemic injection of Fms-related tyrosine 3 ligand (FLT3L) together with the intratumoral injection of polyinosinic:polycytidylic acid (poly I:C) led to expansion and activation of cDC1s and protected mice from rechallenge, while increasing the response to PD-L1 blockade [46]. Importantly, in mouse models, CD103^+^ cDC1s possess the unique capability to transport tumor antigens to lymphoid structures and then to prime CD8^+^ T cells. Accordingly, in human melanoma metastases, cDC1 gene signatures (including THBD, CLEC9A and XCR1) and cytokine profiles such as CXCL9 and CXCL10 correlate with CD8^+^ T cell signatures [44, 46–49].

The cDC1-mediated anti-tumor immunity is limited by factors such as tumor-derived granulocyte colony-stimulating factor (G-CSF), which inhibits cDC1 development through the suppression of IRF8, as well as by hepatitis A virus cellular receptor 2 HAVCR2 (better known as TIM-3), which controls the DNA uptake into, and the cGAS/STING dependent expression of T cell–recruiting chemokines (CXCL9 and CXCL11) by, intratumoral DC [50, 51]. Moreover, in mice, T cell immunoglobulin and mucin domain containing 4 (TIMD4, better known as TIM4), the phosphatidyl serine receptor, facilitates antigen uptake by tissue-resident lung cDC1s, thus driving tumor immunosurveillance [52]. In human lung adenocarcinoma, TIM4 expression correlated with PD-1 treatment responses [52].

The ability of cDC1s to migrate to, and infiltrate, tumors is essential for coordinating immune responses at the site of the tumor, as well as in tertiary lymphoid structures or lymph nodes. The recruitment of cDC1s to tumors is controlled by chemotactic factors produced within the tumor microenvironment, including natural killer (NK) cell-derived chemokines such as CCL5 and XCL1 [40]. Consistently the recruitment of cDC1s to tumors can be increased by the transgenic expression in the malignant cells of FLT3L and XCL1, the chemotactic ligand for the cDC1-specific receptor XCR1 [53]. In patients with metastatic skin cutaneous melanoma, breast cancer, and cervical squamous carcinoma, expression of CCL5 and FLT3L correlated with cDC1 signatures and was associated with better survival [54].

Cancer immune evasion can occur through tumor-derived prostaglandin E2 (PGE2) that impairs cDC1 function as well as tumor-secreted gelsolin that reduces CLEC9A binding to dead cell fragments, thus affecting cDC1-mediated cross-presentation [40, 55]. In several types of cancer including hepatocellular carcinoma, head and neck squamous cell carcinoma, stomach adenocarcinoma and ovarian cancer, overall patient survival appears to be favored by low levels of soluble gelsolin and higher levels of CLEC9A present in the tumor bed [55, 56]. Of note, the loss of secreted gelsolin correlated with enhanced responses to chemotherapy, targeted therapy and radiotherapy, consistent with the notion that immunogenic cell death (ICD) induces T cell-dependent anticancer immunity.

## A novel screening system for the identification of cDC1 activators

**Fig. 2:**
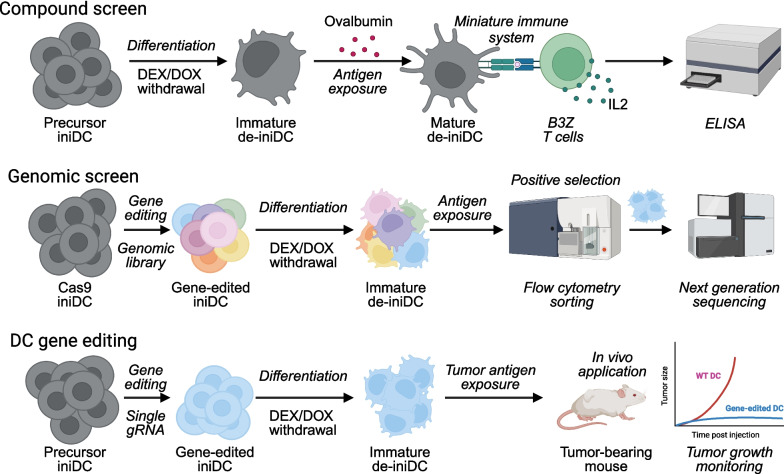
Principles of the ini-DC/de-ini-DC screening system. Chemical compounds are screened using iniDC differentiated into immature de-iniDC upon withdrawal of dexamethasone (DEX) and doxycycline (DOX). De-iniDC are pulsed with chicken ovalbumin before coculture with B3Z T cell hybridoma cells in a sort of miniature immune system. TCR engagement by B3Z cells results in the production of interleukin-2 (IL2) that can be measured by means of an enzyme-linked immunosorbent assay (ELISA). The genome is screened by using a pooled and barcoded guidance RNA (gRNA) library together with iniDC that stably express the CRISPR-CAS9 nuclease. Upon antigen exposure mature antigen-presenting cells are enriched by immunostaining and flow cytometry. Selected cells are further subjected to next generation sequencing for the identification of gRNAs that induce a gain-of-function phenotype. Single CRISPR RNA gene-edited cells are cloned, differentiated and then employed for DC immunotherapy in vivo. (Created with BioRender.com)

We recently developed a cDC1-based screening system that allows for the phenotypic identification of inhibitory immune checkpoints that, when blocked, increase the efficacy of cDC1-mediated antigen cross-presentation. This screening system consists of conditionally induced immortalized dendritic cells (iniDC) precursors derived from C57Bl/6 mice that express the SV40 large T cell antigen under the control of a TET-on promoter and that can be amplified and continuously cultured by conventional cell culture in the presence of dexamethasone (DEX) and doxycycline (DOX). DEX and DOX activate the expression of the SV40 large T cell antigen, leading to the inhibition of RB transcriptional corepressor 1 (RB1) and tumor protein P53 (TP53), hence facilitate the retention of cells in an immortal precursor state. Withdrawal of DEX and DOX triggers the de-induction of RB1 and TP53 expression and thus drives the de-immortalization of the cells, allowing for their differentiation into immature DC (de-iniDC) [13, 57, 58]. Immature de-iniDC are endowed with cDC1-like characteristics such as the pinocytosis of extracellular proteins. As a result, de-iniDC become susceptible to apoptosis induction by cytochrome c (*CYTC*) present in the extracellular space [59, 60]. Moreover, de-iniDC become capable of antigen uptake, processing and peptide presentation by MHC class I molecules to CTLs. In our screening system, we pulsed de-iniDC with chicken ovalbumin (OVA) protein before coculture with B3Z hybridoma cells that express a transgenic T-cell receptor (TCR) specific to the H2-K^b^ MHC class I-restricted OVA-derived SIINFEKL peptide. TCR engagement by B3Z cells results in the production of interleukin-2 (IL2) that can be assessed by means of a conventional enzyme-linked immunosorbent assay (ELISA) [60] (Fig. 2).

A genome-wide CRISPR/Cas9 screen for gain-of-function phenotypes increasing DC-mediated cross-presentation that employed gene-edited iniDC revealed that B-cell lymphoma 2 (BCL2) acts as an endogenous checkpoint to suppress cDC1-mediated tumor immunosurveillance. Genetic or pharmacological inhibition of BCL2 resulted in cDC1- and CTL-dependent effects against solid cancers that were further enhanced by PD-1 blockade [60]. In this setting, the cDC1-dependent regression of orthotopic lung cancers and fibrosarcomas by pharmacological BCL2 inhibitors such as venetoclax and navitoclax was independent of cancer cell-intrinsic mechanisms, based on two sets of observations. First, the malignant cells did not respond to BCL2 inhibition in vitro. Second, malignant cells evolving in immunodeficient (cDC1 or T cell-depleted) mice failed to respond to BCL2 inhibition as well [60]. Consistently reinfusion of de-iniDC reversed immunosuppression in mice lacking *Batf3* and then reactivated venetoclax-mediated anticancer effects. Moreover, the treatment with BCL2 inhibitors was shown to induce the activation of cDC1s detectable in circulation, both in mice and in patients, altogether underlining that BCL2 acts as a cDC1-specific immune checkpoint that restrains tumor immunosurveillance [60, 61].

Furthermore, drug screening based on de-iniDC led to the discovery of drugs that can stimulate cDC1 function. Thus, Toll-like receptor 3 (TLR3) agonists were found to enhance the function of cDC1s lacking formyl peptide receptor 1 (FPR1) in a context in which they have no major effect on WT cDC1s. Indeed, the TLR3 agonists poly: IC and TL-532 are capable of restoring deficient immunogenic chemotherapy responses in *Fpr1*^−/−^ mice through their immunostimulatory action [13, 57, 62]. Moreover, the *Streptomyces-*derived antibiotic ikarugamycin acts as a potent stimulator of antigen presentation by WT de-iniDC [63]. Mechanistically, ikarugamycin inhibits hexokinase 2, leading to DC activation, as indicated by the increased expression of the activation markers CD40, CD80, and CD86. Moreover, ikarugamycin enhanced the capacity of de-iniDC and bone marrow-derived DC (BMDC) to present antigens to B3Z as well as to primary mouse T cells in vitro. In tumor-bearing mice, ikarugamycin synergized with oxaliplatin-based immunogenic chemotherapy and further augmented T cell-mediated anticancer immunity. The ikarugamycin-mediated anticancer effects were lost in T cell-deficient mice, underscoring that they are mediated by a cellular immune response [63].

Altogether, the aforementioned results underline the versatility of our cDC1-based screening system and its utility for large-scale screening campaigns. The possibility of employing gene-edited or pharmacologically enhanced cDC1 for functional in vitro and in vivo assays offers an advantage over alternative screening approaches that might be decisive for the development of future combination regimens against cancer.

## Concluding remarks

Here we summarized findings underlining the crucial role of cDC1s in orchestrating anti-tumor immune responses. Each of the steps in the cascade, namely (1) attraction of cDC1 precursors into the tumor bed, (2) their local differentiation/activation, (3) uptake of tumor antigens by cDC1s and (4) antigen presentation to effector T cells, can be influenced by various mechanisms within the tumor microenvironment. We anticipate that the detailed mechanistic comprehension of these interactions will be important for the development of future cancer therapeutics and cell therapies. Drug screening strategies based on the use of cDC1s can lead to the identification of a novel class of targetable immune checkpoints that operate at the level of cDC1s rather than T cells. The clinical efficacy of ICD has been largely confirmed in clinical trials [64–66] and the combination of ICD-inducing therapy with the functional enhancement of cDC1s promises to stimulate optimal and specific anticancer immunity [67–74]. On theoretical grounds, such combination regimens involving both ICD inducers and cDC1-targeted immune checkpoint inhibitors could be used to sensitize cancer patients to subsequent blockade of the PD-1/PD-L1 interaction or other T cell-targeted immunotherapies. Future clinical trials must evaluate this prospective (Table 1).

**Table 1:** cDC1 cells in cancer immunity

Cancer type	Study	Finding	References
Bladder cancer	Preclinical	cDC1 and CD8^+^ T cells confer immune surveillance and responses to intravesical CD40 agonism	[75]
Breast cancer	Preclinical	Anti-TIM-3 antibody improved response to paclitaxel chemotherapy was cDC1 dependent	[45]
Breast cancer	Correlative	Gene signatures of cDC1 were associated with increased overall survival	[40]
Breast and pancreatic cancer	Preclinical	Tumor-produced granulocyte-stimulating factor downregulated IRF8 in cDC progenitors and interrupted cDC1 development	[50]
Breast cancer (LBC, TNBC)	Correlative	Gene signatures of cDC1 are associated with increased overall patient survival	[39]
Breast cancer	Preclinical	cDC1 interferon signaling was required for T-cell mediated protective responses to breast cancer	[76]
Fibrosarcoma	Preclinical	Rejection of tumors was impaired in cDC1 deficient mice	[77]
Fibrosarcoma	Preclinical	Lack of CD103^+^ DC within the tumor microenvironment dominantly resists the effector phase of an anti-tumor T cell response, contributing to immune escape	[44]
Hepatocellular carcinoma	Preclinical	CD47 blockade enhanced tumor DNA uptake by cDC1 and stimulated the cGAS-STING-dependent infiltration of NK cells in liver cancer	[78]
Liver-engrafted tumors	Preclinical	Depletion of cDC1 in established tumors suppressed immunotherapy efficacy of anti-PD-1 and/or anti-CD137 as well as adoptive T-cell therapy	[79]
Lung cancer	Prognostic and in vitro	cDC1s cross-present human tumor antigen after uptake of necrotic lung cancer cells	[80]
Lung carcinoma and melanoma-induced lung metastasis	Preclinical	Lung tumor development led to the accumulation of regulatory CD103^lo^CD11b^+^ DC and a reduced proportion of cDC1	[81]
Non-small cell lung cancer (NSCLC)	Preclinical	Paucity of cDC1s contributes to reduced antitumor immunity	[82]
Melanoma	Preclinical	Recruitment of cDC1s into tumors was necessary for a CD8^+^ T cell responses	[83]
Melanoma	Preclinical	Efficacy of immunomodulatory anti-CD137 and anti-PD-1 immunotherapy required cDC1	[84]
Melanoma	Preclinical	cDC1 transported antigens to lymph nodes and primed CD8^+^ T cells and promoted anti-tumor effects upon PD-L1 ICI. Combined FLT3L and poly I:C therapy enhanced tumor responses to checkpoint and BRAF blockade	[46]
Melanoma	Preclinical	cDC1 enhanced activation of TCR-engineered T cells	[85]
Melanoma	Predictive	cDC1 among total antigen-presenting cells predicted patient responsiveness to anti-PD-1 therapy	[86]
Melanoma and osteosarcoma	Preclinical	Vaccination with poly I:C-activated and tumor antigen-loaded cDC1s enhanced tumor infiltration of tumor antigen-specific and interferon-γ^+^ CD8^+^ T cells, and suppressed tumor growth	[87]
Melanoma	Preclinical	Administration of Fms-related tyrosine 3 ligand (Flt3L) plus polyI:C and anti-CD40 resulted in an increase of activated cDC1 treated tumors and delayed tumor growth	[88]
Melanoma	Correlative	Human CD141^+^ cDC1 from blood are impaired in patients with advanced melanoma	[89]
Melanoma	Preclinical	Inhibition of the mevalonate pathway in cancer cells triggers cDC1-mediated anticancer immunity	[90]
Melanoma, colorectal cancer	Preclinical	Therapeutic efficacy dead cell antigen-loaded cDC1s was synergistic with anti-PD-1 therapy	[91]
Melanoma, colorectal carcinoma; several human cancer types	Preclinical; prognostic	FLT3LG and CCL5 or CCR5 gene expression signatures correlate with an enhanced cDC1 signature and a favorable overall survival in patients with cancer	[54]
Multiple human tumor biopsies	Correlative	Abundance of cDC1 transcripts correlated with clinical outcome	[92]
Ovarian cancer	Preclinical	PD-1 blockade enabled tumor-associated cDC1s to promote disease clearance	[93]
Ovarian cancer (OvC) and prostate cancer (PrC)	Correlative	cDC1s are reduced in patients with OvC, and are quantitatively and qualitatively impaired in patients with OvC or PrC	[56]
Pancreatic ductal adenocarcinoma (PDAC)	Preclinical	PDAC antigen-loaded cDC1s used as a vaccine, rendering PDAC sensitive to ICI with curative outcome	[94]

## Data Availability

Not applicable.

## Abbreviations

APC: Antigen presenting cell
BMDC: Bone marrow-derived DC
cDC1: Conventional type 1 dendritic cells
CTL: Cytotoxic T lymphocyte
DAMP: Damage associated molecular pattern
DEX: Dexamethasone
DC: Dendritic cells
DOX: Doxycycline
ELISA: Enzyme-linked immunosorbent assay
ICD: Immunogenic cell death
ICI: Immune checkpoint inhibitor
iniDC: Induced immortalized dendritic cells
poly I:C: Polyinosinic:polycytidylic acid
PRR: Pattern recognition receptor
pDC: Plasmacytoid DC
TCR: T-cell receptor
